# Anaemia among adults in Kassala, Eastern Sudan

**DOI:** 10.1186/1756-0500-5-202

**Published:** 2012-07-10

**Authors:** Tajeldin M Abdallah, Ishag Adam, Mutaz A Abdelhadi, Mohammed F Siddig, AbdelAziem A Ali

**Affiliations:** 1 Faculty of Medicine, Kassala University, Kassala, P. O Box 496, Sudan; 2 Faculty of Medicine, University of Khartoum, P.O. Box 102, Khartoum, Sudan; 3 Ministry of Health, Kassala, P.O. Box 74, Kassala, Sudan; 4 Department of Obstetrics and Gynecology, Faculty of Medicine, Kassala University, P.O. Box 496, Kassala, Sudan

## Abstract

**Background:**

The increased heterogeneity in the distribution of social and biological risk factors makes the epidemiology of anaemia a real challenge. A cross-sectional study was conducted at Kassala, Eastern Sudan during the period of January — March 2011 to investigate the prevalence and predictors of anaemia among adults (> 15 years old).

**Findings:**

Out of 646, 234 (36.2%) adults had anaemia; 68 (10.5%); 129 (20.0%) and 37 (5.7%) had mild, moderate and severe anaemia, respectively. In logistic regression analyses, age (OR = 1.0, CI = 0.9–1, *P* = 0.7), rural vs. urban residency (OR = 0.9, CI = 0.7–1.3, *P* = 0.9), female vs. male gender (OR = 0.8, CI = 0.6–1.1, *P* = 0.3), educational level ≥ secondary level vs. < secondary level (OR = 1.0, CI = 0.6–1.6, *P* = 0.8) and Hudandawa vs. non-Hudandawa ethnicity (OR = 0.8, CI = 0.6–1, *P* = 0.1) were not associated with anaemia.

**Conclusion:**

There was a high prevalence of anaemia in this setting, anaemia affected adults regardless to their age, sex and educational level. Therefore, anaemia is needed to be screened for routinely and supplements have to be employed in this setting.

## Findings

### Background

Anaemia is a major public health problem, especially in developing countries [1]. It is common in adult and the prevalence of anaemia is increasing with advancing age [2]. There is however, a significant variation in prevalence of anaemia, both within and between countries, necessitating a need for local data for preventive measures. Anaemia is associated with adverse outcomes among adult such as reduced quality of life, depression, increased disability, higher risk of Alzheimer disease and increased risk of mortality [3,4]. Anaemia is a multifactorial condition and the increased heterogeneity in the distribution of social and biological factors with advancing age makes the epidemiology of anaemia a real challenge [5]. Epidemiology of anaemia is important for deciding the control strategies. Thus, studies investigating these parameters are vital and of great interest, so as to provide health planners and caregivers with fundamental guidelines for the implementation of preventive measures. Therefore, the aim of this work was to investigate the prevalence and predictors of anaemia among adults in Kassala, Eastern Sudan.

## Material and Methods

This was a cross sectional community- based survey of adults (>15 year old) residents in Kassala, Eastern Sudan in the period of January through March 2011. Kassala town situated at mean altitudes of 496 meters above sea level, with population of 298529 inhabitants, 182980 of these are > 15 year old. Six hundred- forty six adults were enrolled in the study and there was no exclusion criterion other than age and pregnancy. After informed consent a pre-tested questionnaire was used to gather socio-demographic data (age, sex, educational level, residence, ethnicity and occupation), medical history of chronic illness such as hepatic disorders, renal disease, thyroid disease, arthritis, blood disorders or diabetes mellitus and awareness of their hemoglobin level. Blood films for malaria were prepared, Giemsa-stained and the number of asexual *plasmodium falciparum* parasites per 200 white blood cells were counted and double-checked blindly by an expert microscopist. Hemoglobin concentration was estimated by HemoCue haemoglobinometer (HemoCue AB, Angelhom, Sweden). Urine was examined by dipsticks for nitrite and considered as indictors for urinary tract infection if it was positive. Microscopic examination of the stool was performed. Anaemia was defined according to the WHO criteria as a hemoglobin concentration lower than 12 gm/dl in women and 13gm/dl in men. Anaemia was defined as mild, moderate and severe when hemoglobin concentration was 9.5.–13 g/dl, 8— 9.4 g/dl and <8 g/dl, respectively.

### Statistic analyses

Data were entered into a computer database and SPSS software (SPSS Inc., Chicago, IL, USA, version 13.0) and double checked before analysis. Means and proportions for the socio-demographic characteristics were compared between the anemic and non-anemic groups using student *t*-test and *x*^2^ test, respectively. Univariate and multivariate analyses were performed. Anaemia was the dependent variable and socio-demographic characteristics with their referral groups were independent variables. Confidence intervals of 95% were calculated and *P* < 0.05 was considered significant. In case of discrepancy between the results of student *t*-test and *x*^2^ test and the results of multivariate analyses, the later was taken as final.

### Ethics

The study received ethical clearance from the Research Board at the Health Research Board at the Ministry of Health, Kassala, Sudan.

## Results

### Patients’ characteristics

Blood test results of 646 adult persons were included in the study. Their mean age was 50.7 ± 2.6 years. Out of these 646 subjected, 321(49.7%) were females, 99 (15.3) were belong to Hudandawa tribe, 274(42.4) had rural residency and 544 (84.2%) had less than secondary educational level.

The mean hemoglobin level was 11.8 ± 2.7 gm/dl. The entire prevalence of anaemia among the surveyed subjects was 36.2% (234/646) and it was 34.2% and 38.3% among male and female, respectively. Anaemia was mild, moderate and severe in 68 (10.5%); 129 (20.0%) and 37 (5.7%) patients, respectively. Almost all (96.3%) of the anaemic subjects perceived themselves as non-anaemic ones. History of tuberculosis and renal insufficiency were reported in 1 and 4 anaemic persons, respectively. While none of the patients had hook worms, two of them had urinary tract infections and one of them had *P. falciparum* malaria.

### Predictors of anaemia

In logistic regression analyses, age (OR = 1.0, CI = 0.9–1, *P* = 0.7), rural vs. urban residency (OR = 0.9, CI = 0.7–1.3, *P* = 0.9), female vs. male gender (OR = 0.8, CI = 0.6–1.1, *P* = 0.3), educational level ≥ secondary level vs. < secondary level (OR = 1.0, CI = 0.6–1.6, *P* = 0.8) and Hudandawa vs. non-Hudandawa ethnicity (OR = 0.8, CI = 0.6–1, *P* = 0.1) were not associated with anaemia, Table 1.

**Table 1:** Epidemiological factors of anaemia among adults in Kassala, Sudan using univariate and multivariate analyses

**P**	OR (95%CI)**	**P**	OR (95%CI)*	
0.7	1.0(0.9–1.0)	0.8	1.0 (0.9–1.0)	**Age (year)**
				**Residence**
	1.0 (ref.)		1.0 (ref.)	Urban
0.9	0.9(0.7–1.3)	0.8	0.9 (0.6–1.3)	Rural
				**Education**
	1.0 (ref.)		1.0 (ref.)	≥ Secondary level
0.8	1.0(0.6–1.6)	0.9	0.9 (0.6–1.5)	< Secondary level
				**Sex**
	1.0 (ref.)		1.0 (ref.)	Male
0.3	0.8 (0.6–1.1)	0.2	0.8(0.6–1.1)	Female
				**Ethnicity**
	1.0 (ref.)		1.0 (ref.)	Non- Hudandawa
0.1	0.8 (0.6–1.0)	0.1	0.9(0.6–1.0)	Hudandawa

* Obtained from univariate logistic regression analysis.

** Obtained from multiple logistic regression analysis.

## Discussion

This study revealed that, anaemia is a major health problem in eastern Sudan. According to the WHO, a severe public health problem exists if the prevalence of anaemia is ≥40% in any group [1]. Therefore, there is a need to re-evaluate or strengthen the current strategies to control anaemia among adults in this setting. In this study anaemia affects adults regardless to their age, sex, educational level and residence. In the same area of the study, we recently observed that pregnant ladies were susceptible to anaemia regardless their age, parity and gestational age [6]. Perhaps, these factors (age, sex) were not found as predictors for anaemia, other factors which were not investigated e.g. tuberculosis and other infectious diseases [7]. The prevalence of anaemia usually increases with age particularly after 60 year and this was explained by increase incidence of chronic illness and poor nutritional status in old people [8,9]. Anaemia was reported in 16%–94% of patients with tuberculosis [10,11]. Like the tuberculosis itself, other infections might lead to anaemia by suppressing erythropoiesis by inflammatory mediators [12]. However, other chronic inflammatory disease such as sexually transmitted diseases, HIV, tuberculosis and inflammatory bowl diseases were not investigated in this study. HIV infection has to be considered as a possible etiologic factor for anaemia among people in Sub-Saharan Africa [13]. Yet, for ethical reasons we could not investigate HIV among these people.

In the current study, only one patient had *P. falciparum* malaria. The area is characterized by unstable malaria transmission [14] and malaria was reported as one of the risk factors for anaemia among pregnant women in Eastern Sudan [15].

The major limitation of our study is that the dynamic of the onset and recovery of anaemia is not yet characterized by using short time (< 1 year) serial blood collection. Ferrucci *et al.,* found that 8% of the older adults with normal hemoglobin level subsequently developed anaemia at 3-year follow up [16]. Also this study didn’t investigate the possible causes of anaemia to facilitate the strategies for the preventive programmes. Recently high level (38.0%) of zinc deficiency was observed among pregnant women in the same area of the study [17].

## Conclusion

There was a high prevalence of anaemia in this setting, anaemia affected adults regardless their age, sex and educational level. Therefore, anaemia is needed to be screened for routinely and supplements have to be employed in this setting.

## Competing interests

The authors declare that they have no competing interests.

## Authors’ contributions

TAM, MAA and MFS carried out the study and participated in the statistical analysis and procedures. AAA and IA coordinated and participated in the study design, statistical analysis and the drafting of the manuscript. All the authors read and approved the final version.
